# Hyperglycemia does not affect tissue repair responses in shear stress-induced atherosclerotic plaques in ApoE−/− mice

**DOI:** 10.1038/s41598-018-25942-3

**Published:** 2018-05-14

**Authors:** Sabrina Hsiung, Anki Knutsson, Jenifer Vallejo, Pontus Dunér, Suvi E. Heinonen, Ann-Cathrine Jönsson-Rylander, Eva Bengtsson, Jan Nilsson, Anna Hultgårdh-Nilsson

**Affiliations:** 1 Department of Experimental Medical Science, Lund University, Lund, Sweden; 2 Department of Clinical Sciences, Lund University, Malmoe, Sweden; 3 Heart Failure Bioscience, Cardiovascular and Metabolic Diseases, IMED Biotech Unit, AstraZeneca, Gothenburg, Sweden

## Abstract

The mechanisms responsible for macrovascular complications in diabetes remain to be fully understood. Recent studies have identified impaired vascular repair as a possible cause of plaque vulnerability in diabetes. This notion is supported by observations of a reduced content of fibrous proteins and smooth muscle cell mitogens in carotid endarterectomy from diabetic patients along with findings of decreased circulating levels of endothelial progenitor cells. In the present study we used a diabetic mouse model to characterize how hyperglycemia affects arterial repair responses. We induced atherosclerotic plaque formation in ApoE-deficient (ApoE−/−) and heterozygous glucokinase knockout ApoE-deficient mice (ApoE−/− GK+/−) mice with a shear stress-modifying cast. There were no differences in cholesterol or triglyceride levels between the ApoE−/− and ApoE−/− GK+/− mice. Hyperglycemia did not affect the size of the formed atherosclerotic plaques, and no effects were seen on activation of cell proliferation, smooth muscle cell content or on the expression and localization of collagen, elastin and several other extracellular matrix proteins. The present study demonstrates that hyperglycemia per se has no significant effects on tissue repair processes in injured mouse carotid arteries, suggesting that other mechanisms are involved in diabetic plaque vulnerability.

## Introduction

Diabetes is associated with a 2- to 3-fold increased risk of cardiovascular disease (CVD) including acute myocardial infarction and stroke, independent of the type of diabetes^1,2^. Most acute cardiovascular events are caused by the rupture of an atherosclerotic plaque^3^. Plaques prone to rupture are characterized by a thin fibrous cap covering a core of lipids and necrotic debris. The mechanisms responsible for the formation of these vulnerable plaques include degradation of fibrous tissue by matrix metalloproteinases (MMPs) released by macrophages in response to inflammatory activation^4^. Since diabetes is associated with a state of moderate chronic inflammation it has been assumed that diabetes increases CVD risk by aggravating inflammation in atherosclerotic plaques^5^. In line with this notion it has been shown that subjects with type 2 diabetes have elevated plasma levels of MMP-7 and −12 and that high levels of these MMPs are associated with an increased risk of development of myocardial infarction^6^. However, there is also evidence that this inflammation is associated with an impaired capacity of vascular tissue repair. Atherosclerotic plaques removed at carotid surgery from patients with diabetes have reduced contents of connective tissue proteins and smooth muscle cell growth factors as compared to plaques from non-diabetics^7^. Subjects with diabetes also have reduced levels of the endothelial progenitor cells (EPCs)^8^ that are important both for maintaining vascular repair and protection against vascular disease^9,10^ as well as for the formation of new blood vessels during healing of cutaneous wounds^11^. Impaired tissue repair thus represents a possible link between chronic ulcers and macrovascular disease in diabetes. Expansion of EPCs is dependent on cleavage of membrane-bound stem cell factor (SCF) on stromal bone marrow cells and the soluble form of SCF subsequently activates the proliferation of EPCs^12^. We have recently shown that subjects with diabetes have lower plasma levels of SCF and that lower levels of SCF are associated with more severe carotid atherosclerosis and an increased risk for development of CV events^13^. SCF-activated vascular progenitor cells have also been implicated in neointima formation following vascular injury^14^. Further support for the notion that diabetes affects arterial repair processes is also supported by observations of increased risk for restenosis following angioplasty^15^.

In the present study we used a diabetic mouse model to investigate how increased glucose levels affect tissue repair responses in atherosclerotic plaques. We used the apolipoprotein E deficient (ApoE−/−) mouse intercrossed with the heterozygous glucokinase mouse (GK+/−). The glucokinase enzyme regulates blood glucose levels through the conversion of glucose to glucose-6-phosphate, the first step in glycolysis. The GK+/− mouse has a reduced glucokinase activity in both the insulin producing β-cells and in the liver resulting in elevated glucose levels and insulin resistance, similar features that are present in human type 2 diabetes^16^. Furthermore, the absence of diabetes-induced increase in lipid levels in the ApoE−/− GK+/− mouse makes it an appropriate model to isolate the effects of hyperglycemia on atherosclerotic plaque development. We used a shear stress-modifying cast to induce both advanced and fibrous carotid atherosclerotic plaques in the ApoE−/− GK+/− and the ApoE−/− mouse. The results show that hyperglycemia does not affect atherosclerotic plaque development in the present mouse model and we propose that other mechanisms than hyperglycemia may be involved in inducing plaque vulnerability in diabetics.

## Results

### Plasma analyses

**Figure 1:**
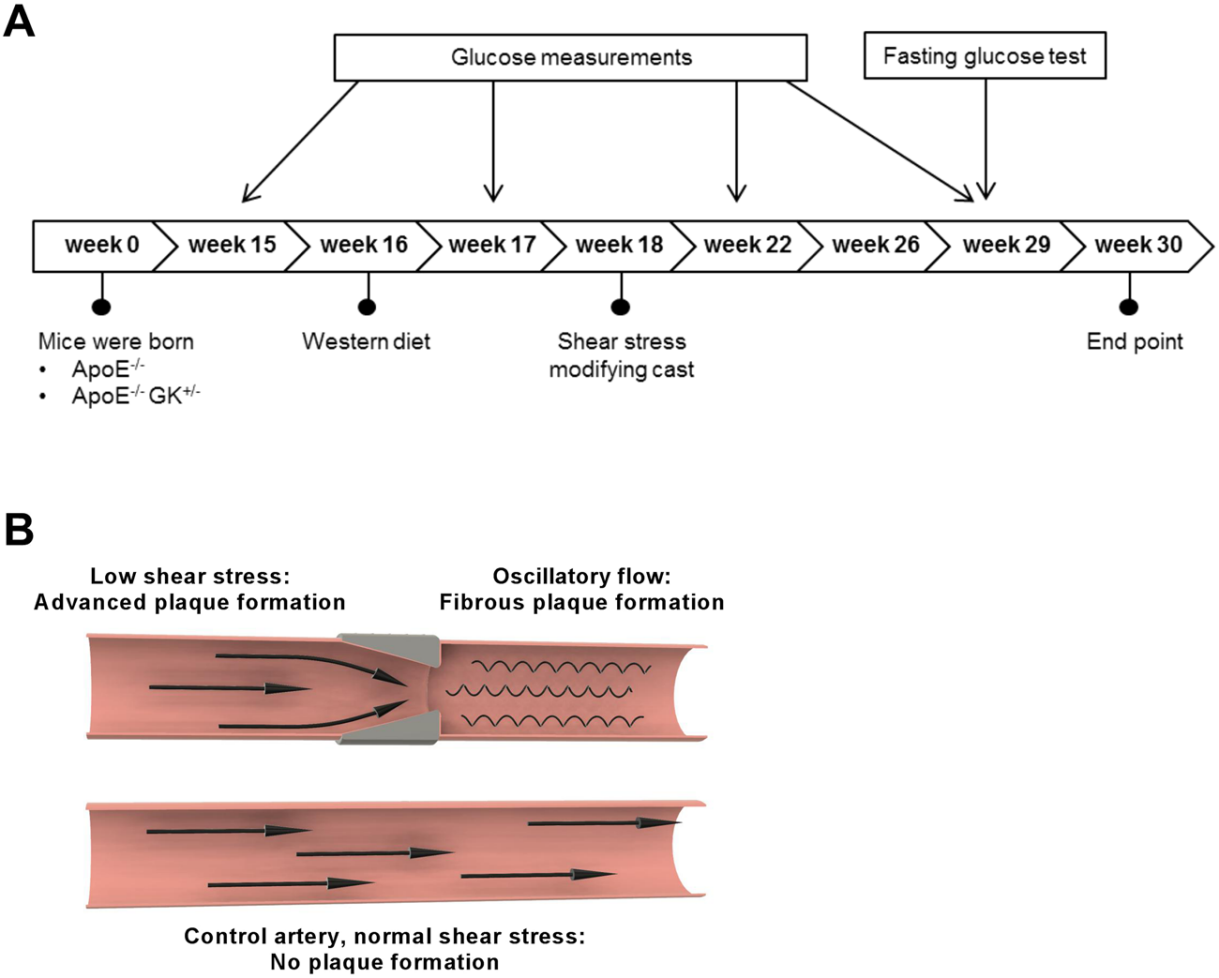
Schematic overview of the experimental mouse model. Timeline and setup over the experiment (**A**) and the shear stress-modifying cast that induces atherosclerotic plaques in the carotid artery (**B**).

**Figure 2:**
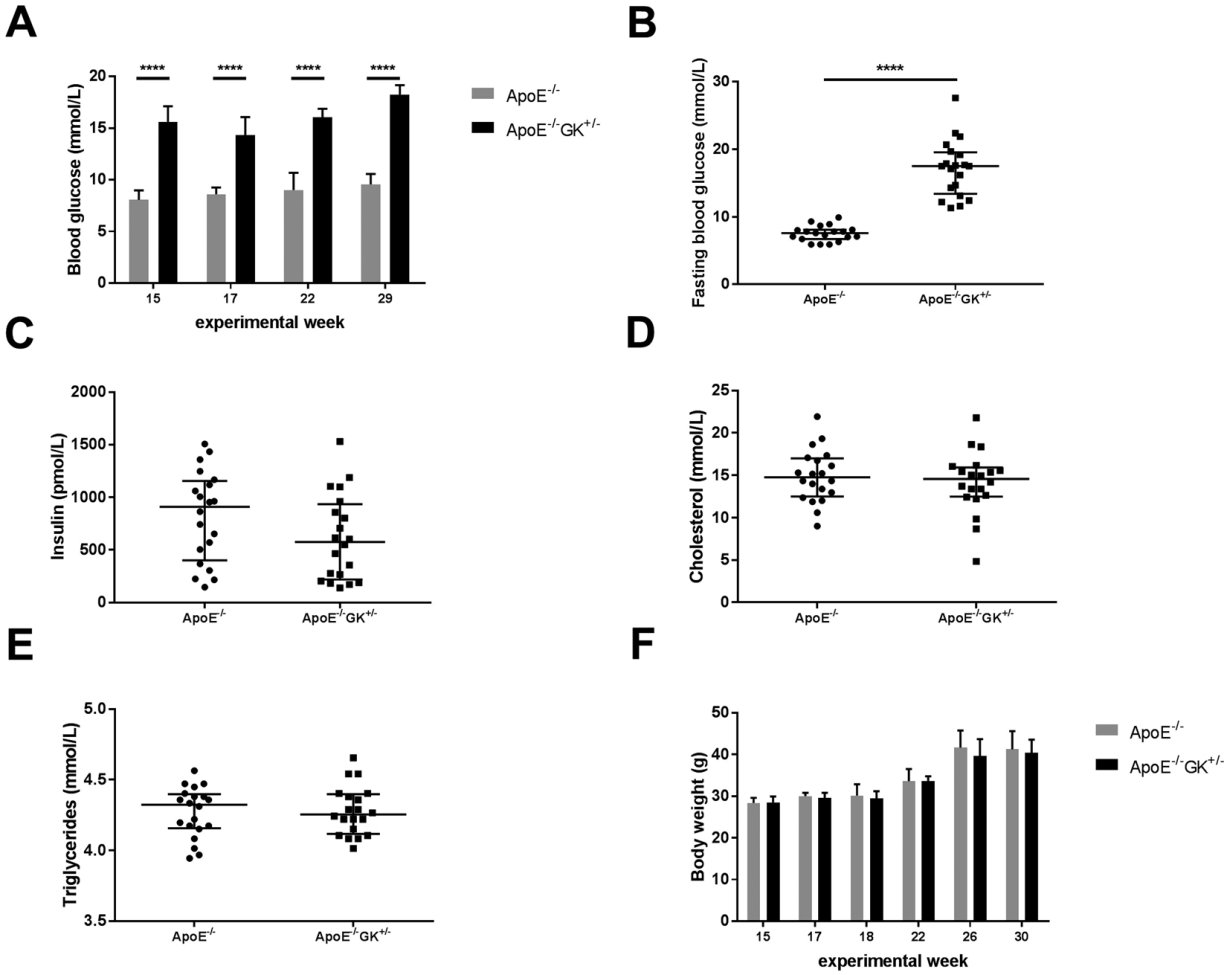
Plasma analyses of metabolic parameters. The ApoE−/−GK+/− mice had significantly elevated blood glucose levels compared to the controls during the entire study (**A**), as well as higher fasting glucose levels (**B**). No difference in insulin levels could be found between the ApoE−/− and ApoE−/−GK+/− mice (**C**). The cholesterol and triglyceride levels were similar between hyperglycemic and normoglycemic mice (**D** and **E**) and the body weight was comparable between the two groups (**F**). ****p < 0.0001.

To assure hyperglycemia in the ApoE−/− GK+/− mice plasma blood glucose levels were measured at different time points; before and after onset of the western diet, at weeks 15 and 17 respectively, and after the surgical placement of the shear stress-modifying cast at week 22 and 29 (Fig. 1A). Mice are considered hyperglycemic at plasma glucose levels between 13.89–16.67 mmol/L (250–300 mg/dl)^17,18^. The ApoE−/− GK+/− mice had significant higher glucose levels compared to the control ApoE−/− mice throughout the whole study (p < 0.0001) (Table 1 and Fig. 2A). A fasting glucose test was also performed at week 29 after a period of 5 hours fasting in the morning (Fig. 1A). The test demonstrated that the ApoE−/− GK+/− mice were hyperglycemic (17.5 mmol/L, IQR 13.4–19.6) and had significant higher glucose levels compared to ApoE−/− mice (7.6 mmol/L, IQR 6.7–8.1) after fasting (p < 0.0001) (Fig. 2B). Insulin levels in plasma were also analyzed and no difference could be found between ApoE−/− GK+/− and ApoE−/− controls (Fig. 2C). The total plasma cholesterol and triglyceride levels were similar between the ApoE−/− and ApoE−/− GK+/− mice (Fig. 2D,E) and no difference in body weight could be found between the two groups (Fig. 2F). The plasma cytokine concentrations for IL-1β, IL-2, IL-4, IL-5, IL-6, IL-10, IL-12(p70), IL-13, IL-17A, MCP-1, IFNy and TNF-alpha also showed no difference between ApoE−/− GK+/− and ApoE−/− mice (data not shown). To investigate how hyperglycemia affects kidney function, plasma creatinine levels were analyzed and showed no difference between the ApoE−/− mice (148 µmol/L, IQR 106.4–204.7) and the ApoE−/− GK+/− mice (125 µmol/L, 72.7–146.6).

**Table 1:** Blood glucose levels in ApoE^−/−^GK^+/−^ and ApoE^−/−^ mice.

Experimental week	ApoE^−/−^GK^+/−^ (n = 20)	ApoE^−/−^ (n = 20)	P-value
week 15	15.4 (IQR 14.7–17.1)	8.1 (IQR 7.5–9.0)	P < 0.0001
week 17	14.3 (IQR 11.8–16.1)	8.6 (IQR 7.8–9.3)	P < 0.0001
week 22	16.1 (IQR 15.0–16.9)	9.0 (IQR 8.5–10.7)	P < 0.0001
week 29	18.3 (IQR 16.0–19.2)	9.6 (IQR 8.7–10.6)	P < 0.0001

Mann-Whitney t-test for nonparametric data was used for comparisons between the two groups. Data is presented as median with interquartile range.

### Elevated plasma glucose levels do not affect plaque size

**Figure 3:**
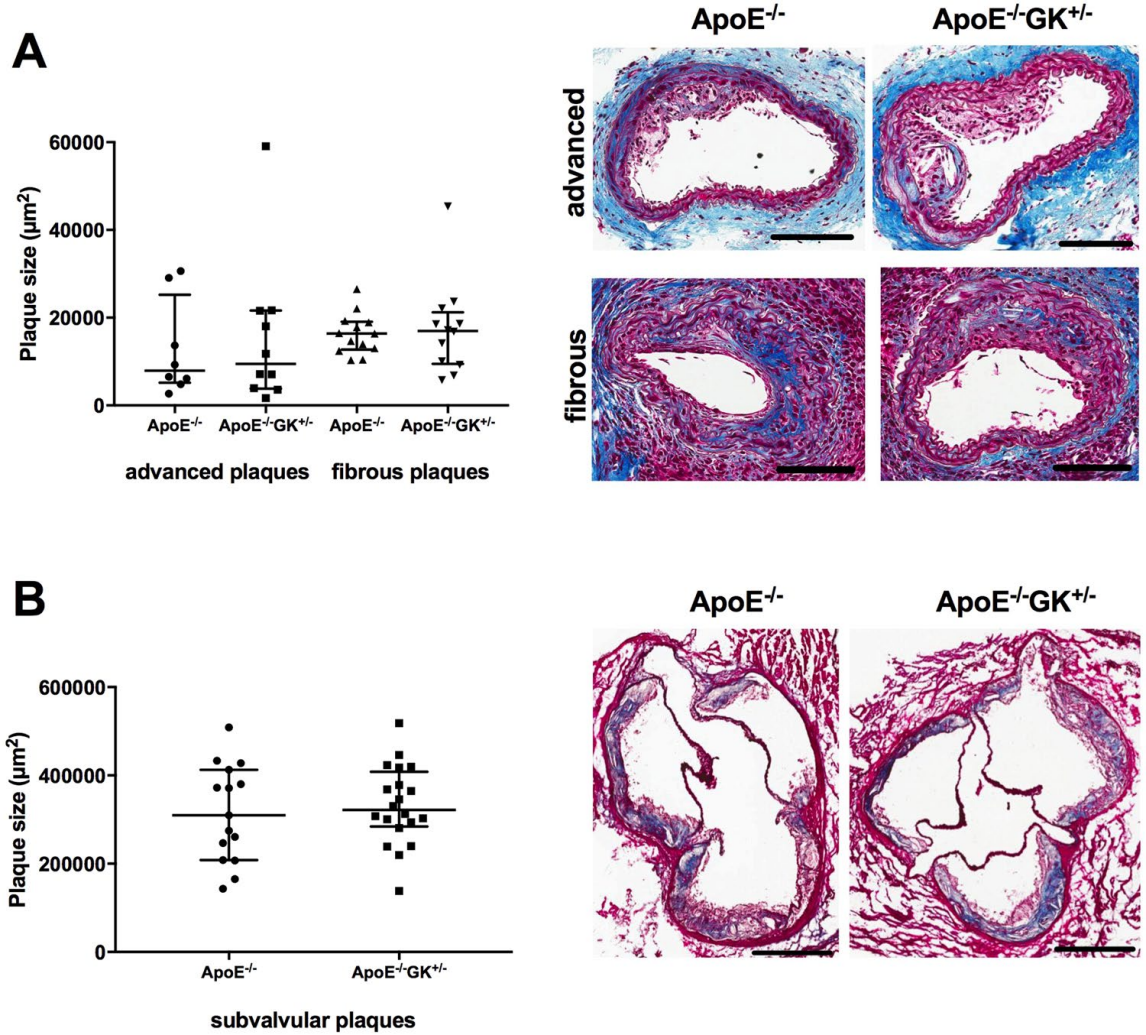
Hyperglycemia does not affect the plaque size. Overview of the induced lesions in the carotid artery showing no difference in advanced or fibrous plaque size between the ApoE−/−GK+/− and ApoE−/− mice (**A**). Visualized by Masson’s Trichrome stain. Scale bars = 100 µm. (**B**) Subvalvular plaques from aortic root sections stained with Masson’s Trichrome showing similar plaque size between the ApoE−/−GK+/− and ApoE−/− mice. Scale bars = 400 µm.

The shear stress-modifying cast resulted in formation of both advanced and fibrous carotid atherosclerotic plaques in the ApoE−/− GK+/− and ApoE−/− mice. No differences in the plaque size could be seen for the advanced or fibrous plaques between the ApoE−/− GK+/− mice and ApoE−/− mice (Fig. 3A). The subvalvular plaque area in aortic root sections was also quantified and there was no difference between the two groups (Fig. 3B).

### Tissue repair responses in plaques are not affected by hyperglycemic conditions

**Figure 4:**
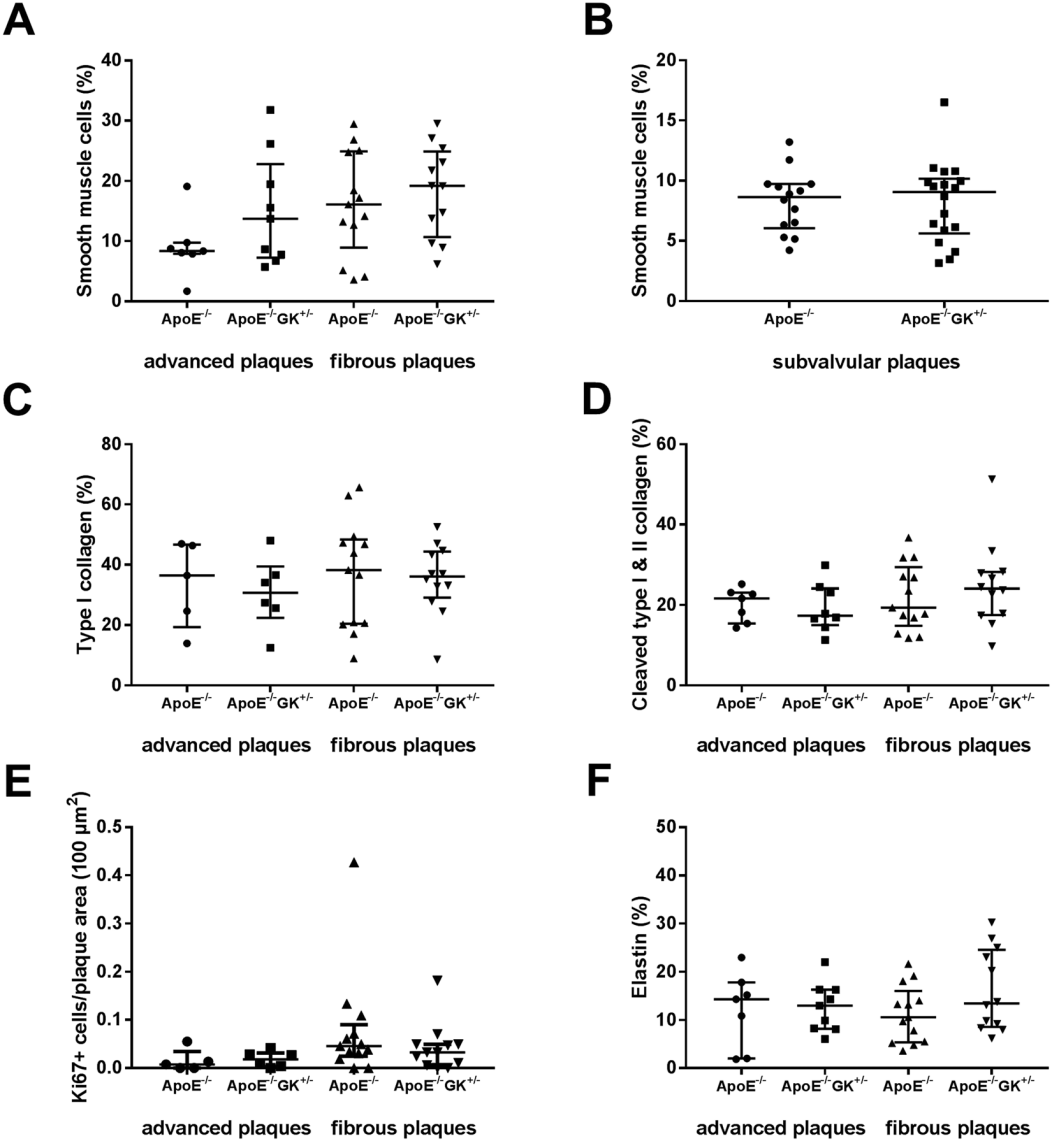
Elevated glucose levels do not affect tissue repair responses. No differences were found in MHC-11 positive smooth muscle cell content in advanced or fibrous plaques (**A**) or in subvalvular plaques (**B**). The type I collagen or cleaved type I and II collagen content in the lesions were not affected in either the ApoE−/− GK+/− mice or the ApoE−/− controls (**C** and **D**). No difference could be seen in the amount of Ki67 + proliferating cells (**E**) or in elastin content (**F**) between the two groups.

Vascular smooth muscle cells (SMCs) have an important role in the growth and stabilization of atherosclerotic plaques. As part of the response to a vascular injury SMCs become activated and change phenotype into synthetic cells that proliferate and synthesize collagen^19,20^. We used the antibody myosin heavy chain 11 (MHC-11) to detect SMCs in the lesions. Impaired glucokinase function did not affect the content of SMCs in advanced plaques (Fig. 4A), fibrous plaques (Fig. 4A) or in subvalvular plaques (4B) compared to the ApoE−/− controls. To analyze the collagen content, composition and degradation in lesions we performed immunohistochemical stainings for type I collagen as well as cleaved type I and II collagen. The results showed that elevated glucose levels did not affect the type I collagen content, nor the amount of cleaved type I and II collagen, for either the ApoE−/− GK+/− mice or the ApoE−/− control mice (Fig. 4C,D). Since activation of SMC proliferation is an important part of the response to a vascular injury the number of actively proliferating (Ki67+) cells was measured in the lesions. No difference in proliferation could be found between the two groups (Fig. 4E). Elastin similarly to collagen confers stability to the plaque^19^. The total elastin content within the plaques was analyzed by immunohistochemistry. The results showed no difference in the elastin content in the lesions between the ApoE−/− GK+/− and the ApoE−/− mice (Fig. 4F).

**Figure 5:**
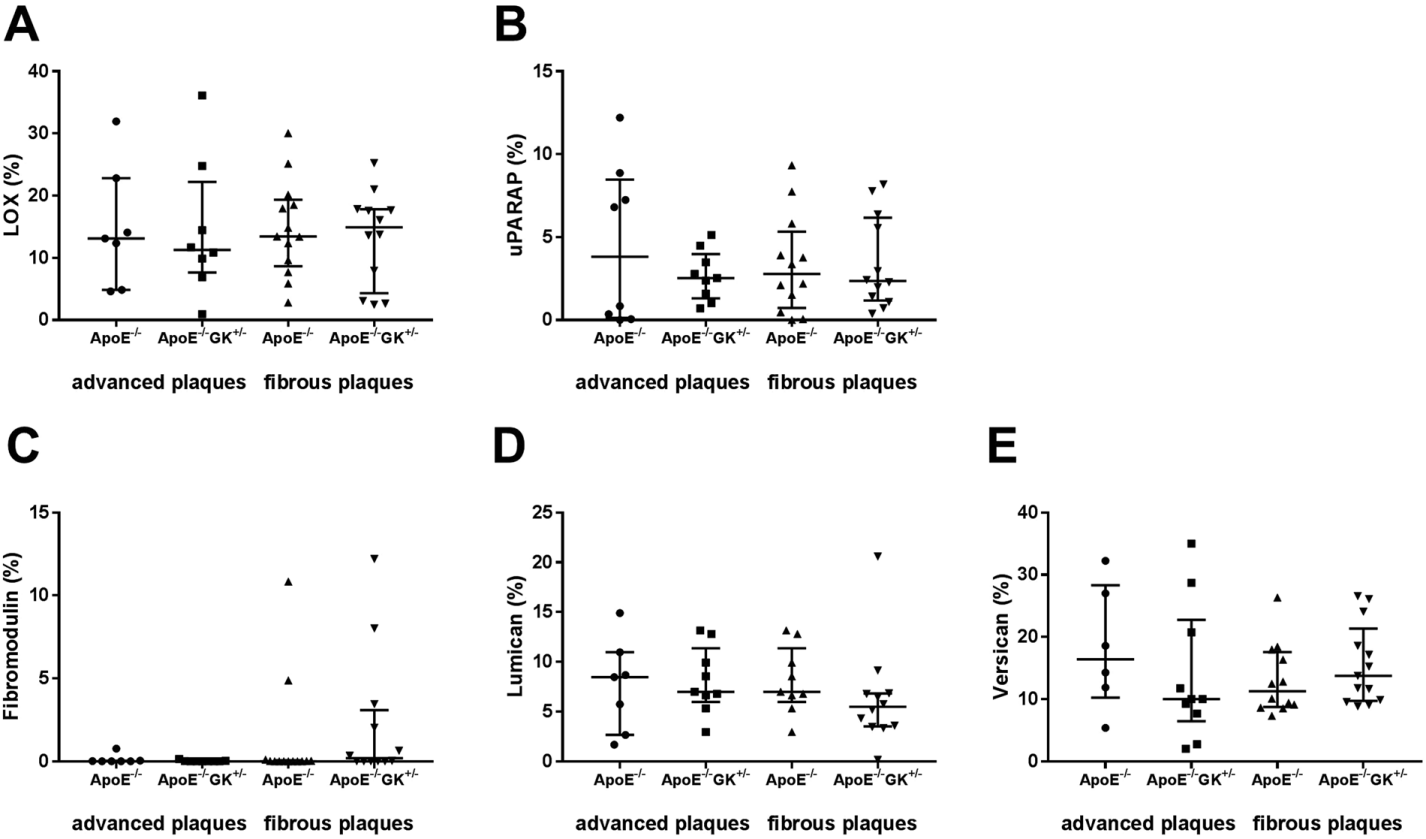
Analyses of the expression of proteins involved in extracellular matrix turnover in the induced carotid lesions. No difference could be observed in the levels of lysyl oxidase (LOX) (**A**), uPARAP (**B**), fibromodulin (**C**), lumican (**D**) or versican (**E**) between the ApoE−/−GK+/− and ApoE−/− mice.

We next analyzed if diabetes affected the expression of a number of different proteins involved in the formation and turnover of the fibrous extracellular matrix. Lysyl oxidase (LOX) is an enzyme that catalyzes the covalent crosslinking of fibers as a post-translational modification of collagens and elastin in the extracellular matrix^21^, while uPARAP is an endocytic collagen receptor with a role in the clearance of collagen^22^. The small leucine-rich proteoglycans lumican and fibromodulin are involved in the regulation of collagen fiber formation^23^ and versican is involved in elastic fiber fibrillogenesis^24^. There were no differences in the expression or location of any of these proteins in the atherosclerotic plaques between the ApoE−/− and ApoE−/− GK+/− mice (Fig. 5A–E).

### The accumulation of inflammatory cells in the atherosclerotic plaque is not affected by hyperglycemia

**Figure 6:**
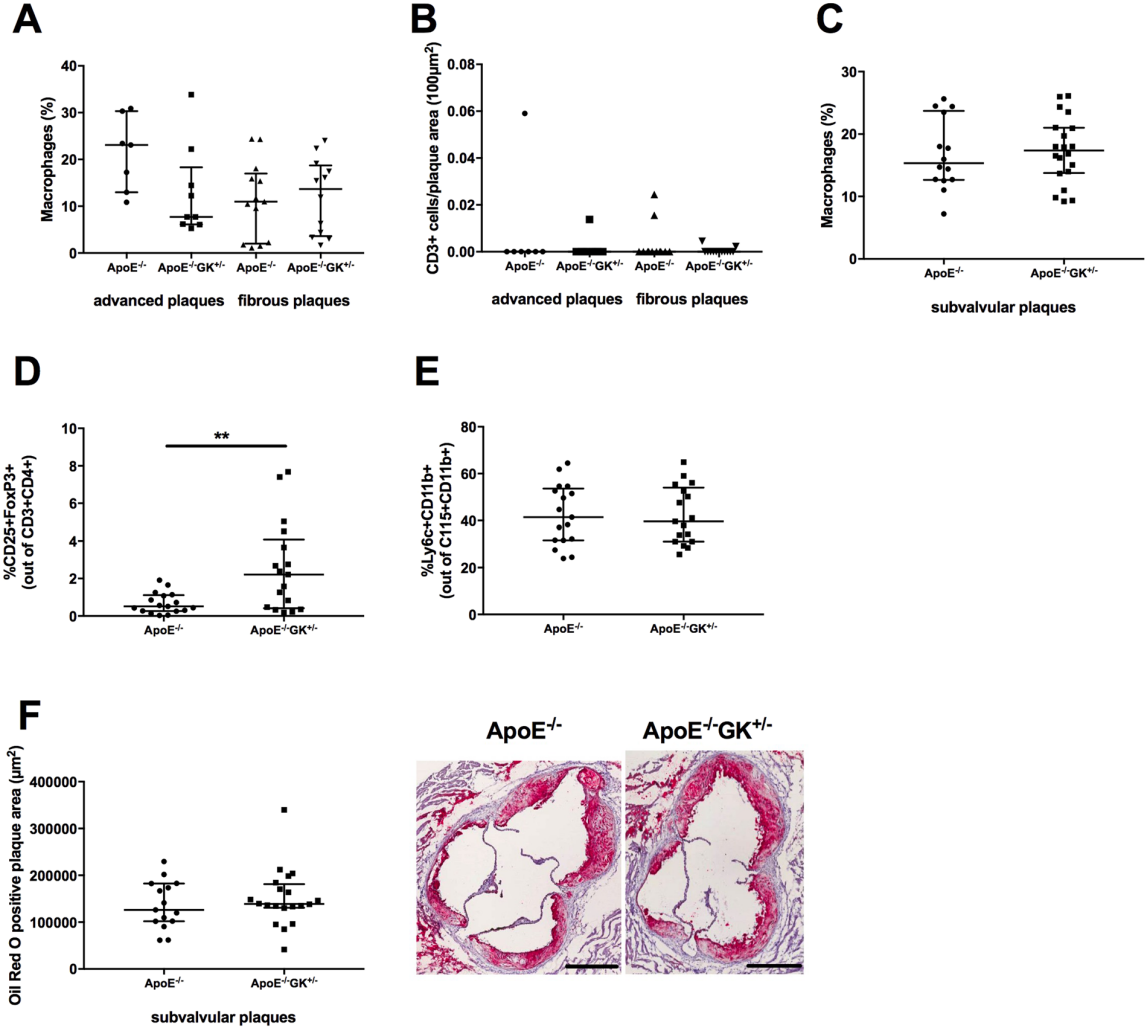
Inflammation is not affected by hyperglycemic conditions in ApoE−/−GK + /− mice. No difference could be found in Mac-2 positive macrophage content in the carotid artery plaques (**A**) or in the subvalvular plaques (**C**). The amount of CD3 positive T-cells in plaques was also similar between the ApoE−/−GK+/− mice and controls (**B**). Increased amount of regulatory T-cells in blood could be observed (**D**), whereas no difference in monocytes could be found (**E**). Subvalvular plaques stained with Oil Red O showing no difference in lipid content between the two groups. Scale bars = 400 µm (**F**). **p < 0.01.

Both macrophages and T-cells are inflammatory cells that promote the atherosclerotic plaque progression. Macrophages destabilize plaques through their release of MMPs and are involved in the formation of lipid-laden necrotic cores, and certain subsets of T-cells promote the atherosclerotic progression by enhancement of the inflammatory environment^25,26^. We analyzed the content of these two cell types in the advanced and fibrous plaques induced by the shear stress-modifying cast, using the macrophage marker Mac-2 and the T-cell marker CD3, and found no difference between the ApoE−/− GK+/− and ApoE−/− mice (Fig. 6A,B). In addition, the amount of Mac-2 positive cells in subvalvular plaques from aortic root sections was analyzed and the result showed no difference between the two groups (Fig. 6C).

The amount of regulatory T-cells (Tregs), which suppress pro-atherosclerotic effects^27^, and monocytes were analyzed in blood through flow cytometry. A significant increase in the Tregs could be found for the ApoE−/− GK+/− mice (2.21%, IQR 0.41–4.08) (p = 0.0085) compared to the ApoE−/− controls (Fig. 6D and Supplementary Figure S1) while the amount of monocytes in blood was similar between the two groups (Fig. 6E and Supplementary Figure S1).

We also measured the lipid content within the carotid artery plaques and the subvalvular plaques with an Oil Red O staining to get another view of the atherosclerotic plaque progression. No difference could be found between the ApoE−/− and ApoE−/− GK+/− mice in the subvalvular plaques (Fig. 6F) or in the carotid artery plaques (data not shown).

### Advanced glycation end products and high mobility group box 1 protein

A chronic hyperglycemic state results in the formation of advanced glycation end products (AGEs) through a nonenzymatic glycosylation process of lipids and proteins^28^. High mobility group box 1 protein (HMGB1) is a nuclear protein that also is associated with high glucose levels and diabetes^29,30^. We analyzed the content of AGEs and HMGB1 in the induced atherosclerotic plaques; however, we did not find any differences between the hyperglycemic and normoglycemic mice (Supplementary Figure S2).

## Discussion

Both type 1 and type 2 diabetes are independent risk factors for CVD in both men and women^2,31^. Diabetes is generally associated with a chronic low-grade systemic inflammation, dyslipidemia, oxidative stress and formation of AGEs that may contribute to the development of macro- and microvascular complications^32–34^. However, the underlying molecular mechanisms for the increased CV risk in diabetics still remains to be fully understood. More recently, impaired tissue repair responses have been proposed as a potential cause for plaque vulnerability in diabetes^7^. To explore the effects of hyperglycemia in advanced and fibrous carotid atherosclerotic plaques, induced by a shear stress modifier (Fig. 1), we used ApoE−/− GK+/− mice with ApoE−/− mice as controls. The impaired function of the glucokinase enzyme in both the pancreas and in the liver results in hyperglycemia and insulin resistance^35^. We analyzed the effects of hyperglycemia on atherosclerotic plaque size and no differences were found in either the carotid artery or in the aortic root for the ApoE−/− GK+/− and the ApoE−/− mice (Fig. 3). Taken together, these results show that hyperglycemic conditions do not affect lesion formation. In line with these findings, Adingupu *et al*. reported that impaired glucokinase function in ApoE−/− mice on a western diet did not result in accelerated atherosclerotic plaque progression^35^.

Atherosclerotic plaque formation involves phases of inflammation, excessive proliferation of SMCs and extracellular matrix (ECM) remodeling^36^. The SMCs normally reside quiescent in a contractile state in the medial layer of the artery where their main function is to regulate vascular tone through contraction and dilation of the vessel wall. However, in response to injury the SMCs switch into a synthetic phenotype and start to proliferate and to produce ECM proteins such as collagen and elastin^20^. In a study comparing symptomatic and asymptomatic endarterectomy specimens from patients with or without diabetes Edsfeldt and coworkers^7^ found that symptomatic plaques in non-diabetics were characterized by higher content of pro-inflammatory cytokines. In contrast, the symptomatic plaques from subjects with diabetes did not contain increased levels of pro-inflammatory cytokines but instead exhibited decreased amounts of collagen and elastin suggesting that the mechanisms responsible for plaque vulnerability may be different in diabetes and involve impaired connective tissue repair. The present study shows that hyperglycemia per se does not affect the expression of collagen or proteins important for collagen assembly, nor the elastin content in injured arteries. We used the SMCs marker MHC-11 to measure the amount of SMCs in plaques from the carotid artery and the aortic root. The results showed that elevated glucose levels did not have an effect on the amount of SMCs in the ApoE−/− GK+/− mice compared with the ApoE−/− mice (Fig. 4). The amount of proliferating cells in the lesions, as measured by Ki67 positive cells, did not differ between hyperglycemic and normoglycemic mice (Fig. 4). Collagen and elastin are ECM proteins produced by the synthetic SMCs and are important in tissue repair and thus essential for plaque stabilization. Immunohistochemical analyses of elastin, type I collagen as well as for cleaved type I and II collagen showed no differences between ApoE−/− GK+/− and ApoE−/− mice (Fig. 4). The expression and content of numerous proteins important for the extracellular turnover and fibrous repair responses including LOX, uPARAP, fibromodulin, lumican and versican were also found to be similar in normo- and hyperglycemic mice (Fig. 5).

To further investigate how hyperglycemia affects atherosclerotic plaque progression in the ApoE−/− GK+/− mice we analyzed the macrophage and T-cell contents in plaques by immunohistochemistry, as well as measured the levels of monocytes and Tregs in blood by flow cytometry. The ApoE−/− GK+/− mice did not differ from the controls when it came to plaque T-cell content (Fig. 6). The amount of macrophages in plaque tissue was also similar between the two groups; however, a significant increase of Tregs in blood was found for the ApoE−/− GK+/− mice (Fig. 6 and Supplementary Figure S1). Tregs exert atheroprotective effects and inhibit inflammation through the release of anti-inflammatory cytokines such as IL-10 and TGF-β^27,37^. However, as there was no other evidence of reduced plaque or systemic inflammation in ApoE−/− GK+/− mice it appears this increase in circulating Tregs was without functional importance. Elevated glucose levels did not affect the lipid content in plaques as quantified by an oil red O staining (Fig. 6). Furthermore, the level of several cytokines in plasma was analyzed showing no difference between hyperglycemic and normoglycemic mice (data not shown). Taken together, the results from our study indicate that hyperglycemia per se does not accelerate inflammatory processes in the ApoE−/− GK+/− mouse. Our results support Edsfeldt *et al*. report demonstrating that inflammation is not enhanced in symptomatic carotid endarterectomy from patients with diabetes^7^ and suggest that plaque vulnerability in diabetics may be caused by other mechanisms than increased inflammation.

Some diabetic rodent models are associated with an unstable phenotype, which can progress and do not reflect the diabetic setting seen in humans^18,38^. Several studies have reported that diabetic animal models are associated with diabetes-induced elevation of plasma cholesterol levels, which makes it hard to separate the effect of hyperglycemia on atherosclerosis per se^39–42^. To rule out the influence of abnormal lipid levels on atherosclerotic lesion progression we used the heterozygous glucokinase knockout apolipoprotein E deficient mouse model that has a consistent diabetic profile. Unlike many other diabetic mouse models these mice have a balanced hyperglycemic profile regardless of the age, and most importantly the effect of a diabetes-induced increase in lipid levels is absent which makes it an appropriate model^35^. To confirm this phenotype we measured the total plasma cholesterol and triglyceride levels and indeed, no difference between the ApoE−/− GK+/− and ApoE−/− mice could be found (Fig. 2). Thus, ApoE−/− GK+/− mice are appropriate as a model to study the effect of hyperglycemia per se on arterial repair and atherosclerotic plaque formation. In this study we found no evidence for an independent role of hyperglycemia in any of these two processes. There are several studies that support our findings that hyperglycemia in itself does not contribute to accelerated atherosclerosis. Al-Mashhadi *et al*. compared atherosclerotic lesions in the thoracic aorta in diabetic hypercholesterolaemic minipigs and controls. The authors found no difference in the atherosclerotic progression such as lesion size or collagen content. In addition, the impaired glucose control did not affect the total plasma cholesterol levels between the two groups which in line with the result from our study^43^. It has also been shown that elevated glucose levels had no effect on the development of atherosclerosis in diabetic Ldlr−/− mice as compared to control mice. Importantly, the total cholesterol levels were also similar between the two groups^44^. In a study by Renard *et al*. it is demonstrated that the initial increase in atherosclerotic lesion area seen in diabetic mice was eliminated when the cholesterol levels were matched to the non-diabetic controls^45^. Diabetic kidney disease is another complication of diabetes that causes an elevation in serum creatinine; which is associated with an increased risk of CVD^46,47^. The creatinine levels were similar between the ApoE−/− GK+/− and ApoE−/− mice, indicating that the elevated glucose levels did not alter the kidney function. Additionally, evidence from a human study where patients with a dysfunction in the glucokinase enzyme, resulting in mild hyperglycemia from birth, showed that they did not distinguish themselves from the control group when it came to the risk for developing microvascular and macrovascular complications. Interestingly these patients also have comparable lipid levels to the average population^48^. Taken together, several studies demonstrate that hyperglycemia itself is not sufficient to exert pro-atherosclerotic effects unless a more severe hyperlipidemia is present, which further strengthen the results in the present study.

In conclusion, we have studied pro-atherosclerotic effects of hyperglycemia in a diabetic atherosclerotic mouse model. The findings from the present study show that an impaired glucose control does not exert any effects on arterial repair responses nor on the atherosclerotic process as such. Thus, we propose that hyperglycemia per se does not affect tissue repair processes in the atherosclerotic plaque.

### Study limitations

In humans cardiovascular complications occur several years or decades after the diagnosis of diabetes. We have investigated the effects of hyperglycemia on atherosclerotic plaque development in a mouse model of type 2 diabetes where the lipid levels are similar between the hyperglycemic and normoglycemic mice. This does not reflect type 2 diabetes in humans that is associated with plasma lipid and lipoprotein abnormalities, and accompanied with an increased risk of cardiovascular disease^49^. The accelerated plaque development seen in experimental ApoE−/− mouse models do not reflect the time frame for plaque development seen in humans, but the features of the atherosclerotic process are similar. In our study, the shear-stress induced plaques were left to develop over a period of 12 weeks during which the ApoE−/− GK+/− were hyperglycemic. We have only analyzed plaques at one time point. Adding earlier or longer time points could identify effects of hyperglycemia on plaque development that has not been observed in the present study.

## Material and Methods

### Animals

All animal experiments were approved and performed in accordance with the Malmoe/Lund regional ethical committee in Sweden. Male ApoE−/− (n = 20) and combined heterozygous glucokinase and ApoE-deficient mice (ApoE−/− GK+/−) mice (n = 20) were attained from AstraZeneca, Sweden^16^.

### Surgical procedure and tissue preparation

Male ApoE−/− and ApoE−/− GK+/− mice were fed an atherogenic western diet (WD) (R638; maize starch, cocoa butter, casein, glucose, sucrose, cellulose flour, minerals, and vitamins; 17.2% protein, 21% fat [62.9% saturated, 33.9% unsaturated and 3.4% polyunsaturated], 0.15% cholesterol, 43% carbohydrates, 10% H_2_O, and 3.9% cellulose fibers) for the duration of 14 weeks starting at the age of 16-weeks. A perivascular shear stress-modifying cast was placed around the right common carotid artery at week 18 to alter the hemodynamic flow. As described by Cheng *et al*., the cast induces atherosclerotic plaques with two different phenotypes^50^. Proximally to the cast, plaques with an advanced phenotype are formed due to the lowered shear stress. Distally to the cast, an oscillatory blood flow induces plaques with a more fibrous phenotype. The surgery was carried out under narcosis with oxygen-carried isoflurane. Buprenorphine was administered subcutaneously at 0.1 mg/kg pre-operation and ∼5 hours post-operation. The mice were sacrificed at 30-weeks of age by an overdose of isoflurane (Fig. 1A,B).

Blood was collected by cardiac puncture and plasma was retrieved by centrifugation at 3000 rpm for 15 min at 4 °C. The mice were perfused with HistoChoice (Amresco, H102–1000ML) and the collected tissue samples were fixed in HistoChoice (Amresco, H102-1000ML). The carotid arteries were either embedded in paraffin or frozen in OCT medium (Sakura, 4583) and sectioned to determine the atherosclerotic plaque burden. The left carotid arteries were used as controls. Hearts were embedded in OCT Cryomount (Histolab Products AB, Gothenburg, Sweden).

### Histological analyses

#### Immunohistochemistry

Paraffin carotid artery sections were cut at 5 μm and frozen aortic roots sections were cut at 8 µm. The Vector Laboratories ImmPress Peroxidase detection kits (Vector Laboratories, MP-7444 and MP-7401) were used according to the manufacturer’s instructions. In short the paraffin embedded sections were deparaffinized and rehydrated. For antigen unmasking the slides were submerged in sodium citrate buffer at pH 6.0 and boiled in the microwave for 20 min. The hydrogen peroxide step was omitted. For lumican, versican and fibromodulin, a Chondroitinase ABC digestion step was performed according to the manufacturer’s instructions (MP Biomedicals; 190334). The frozen aortic root sections were dried at 37 °C for one hour and thereafter washed in PBS prior to staining. All the sections were blocked for 20 min using the provided blocking solution. The carotid artery sections were incubated over night at 4 °C with anti-Lox EPR4025 (abcam 174316; diluted 1:1500), anti-elastin (abcam 21610; whole serum diluted 1:1000), anti-Fibromodulin (gift from Anders Aspberg; diluted 1:500), cleaved collagen C1,2 C (Col 2 3/4Cshort) (IBEX 50-1035; serum diluted 1:200), anti-collagen type I (abcam 34710; 2 µg/ml), anti-lumican (abcam 168348; 5 µg/ml), anti-versican (AB1032 Millipore; 10 µg/ml), anti-uPARAP (abcam70132; 2 µg/ml), anti-Ki67 antibody (abcam 16667; diluted 1:200), anti-AGE (abcam 23722; diluted 1:300), anti-HMGB1 [EPR3507] (abcam 79823; diluted 1:1000), anti-Mac-2 (Cederlane; CL8942AP; 0,33 µg/ml), anti-smooth muscle myosin heavy chain 11 (abcam 125884; 5 µg/ml) or anti-CD3 (abcam 11089; 5 µg/ml). The frozen aortic root sections were incubated over night at 4 °C with anti-Mac-2 (Cederlane; CL8942AP; 0.33 µg/ml) or anti-smooth muscle myosin heavy chain 11 (abcam 125884; 5 µg/ml). The secondary antibody provided in the kit, anti-rabbit or anti-rat, was applied for 30 min. ImmPACT DAB peroxidase substrate kit (Vector Laboratories, SK-4105) was used until the desired intensity was developed. Mayer’s Hematoxylin was used as a counter stain (Histolab, 01820) for 3 minutes. The slides were dehydrated, cleared in xylene and mounted using Pertex (HistoLab, 008119).

#### Lipid stainings

Frozen carotid artery sections were cut at 7 µm and frozen aortic root section were cut at 8 µm. The sections were washed in PBS and incubated in 60% isopropanol before they were stained with 0.3% Oil red O for 20 minutes. The sections were then rinsed in 60% isopropanol, washed in PBS, counterstained with Mayer’s Hematoxylin (HistoLab, 008119) and mounted with Mountquick Aqueous (HistoLab, 00960).

### Plasma analyses

For blood glucose analysis blood samples were drawn from the saphenous vein at different time points and measured with a glucometer (Bayer Diabetes Care, Contour XT EAN: 5016003725500); on chow diet at week 15, after onset of the atherogenic western diet at week 17 and after the cast placement at week 22 and 29. A fasting glucose test was also performed after 5 hours fasting in the morning at week 29 (Fig. 1A).

The Mercodia Mouse Insulin kit (Mercodia, 10-1247-01) was used for measurement of insulin level in plasma samples. The Infinity^TM^ Cholesterol (ThermoScientific, TR13421), Infinity^TM^ Triglycerides (ThermoScientific, T22421) and Infinity^TM^ Creatinine (ThermoScientific, TR35121) kits were used to quantify the total plasma cholesterol, triglycerides and creatinine levels together with the standard Data-Cal^TM^ Chemistry Calibrator (ThermoScientific, TS106500). Bio-Plex multiplex technology (Merck Millipore) was used to quantify the plasma cytokine concentrations of IL-1β, IL-2, IL-4, IL-5, IL-6, IL-10, IL-12(p70), IL-13, IL-17A, MCP-1, IFNy and TNF-alpha. All analyses were performed according to the manufacturer’s instructions.

### Flow cytometry

Blood cells were washed twice with staining buffer (Biolegend, San Diego, CA, USA) at 800 rcf for 5 min at 4 °C. Afterwards, they were stained with fluorochrome-conjugated antibodies at a concentration of 1 million cells after blocking of FC receptor (CD16/32, Biolegend, San Diego, CA, USA) for 5 minutes. The following antibodies were used: FoxP3-PE, CD3-PE/Cy7, CD25-APC, CD4-PB, Ly6c-PEcy7, CD115-APC and CD11b-PB all from Biolegend (San Diego, CA, USA). The cells were stained for 30 min on ice in the dark. Erythrocytes were lysed with lysing buffer (BD Pharm Lyse^TM^, BD Biosciences, Stockholm, Sweden) for 15 min on ice in the dark. Before intracellular staining for FoxP3, cells were washed to remove unbound extracellular antibodies and incubated with Foxp3 Fixation/Permeabilization buffers (eBioscience, San Diego, CA, USA) for 30 min on ice. Cells were blocked again with CD16/32 prior to incubation with intracellular FoxP3 for 30 min. Cells were resuspended in PBS buffer containing 1% BSA and 0.5 mM EDTA. Cells were run in Gallios flow cytometer (Beckman Coulter, High Wycombe, UK) and analyzed with FlowJo software (Tree Star, Inc. Ashland, OR, USA).

### Morphometric measurements and statistics

Biopix iQ software 3.3.1 (BioPixAB, Gothenburg, Sweden) was used for all morphometric measurements. The absolute carotid and subvalvular lesion areas were calculated as the mean value from three different sections per lesion. The quantifications of elastin, lox, uPARAP, versican, lumican, fibromodulin, AGE, HMGB1, cleaved type I and II collagen, collagen type I, Mac-2 and smooth muscle heavy chain 11 were calculated as percentages of the plaque areas. Proliferation was calculated as the number of Ki67 positive cells per 100 µm^2^ plaque area and the amount of T-cells was calculated as the number of CD3 positive cells per 100 µm^2^ plaque area. Lipid content in the carotid artery plaques and the subvalvular plaques was calculated as the mean percentage of the plaque area from three subsequent sections. Data is presented as median with interquartile range. Statistical significance was determined with the nonparametric Mann-Whitney U test for skewed data. We used GraphPad Prism version 7 for all of our analyses (GraphPad Software, San Diego California USA).

## Electronic supplementary material

Supplementary information
