# Supplementary material for: Hyperglycemia does not affect tissue repair responses in shear stress-induced atherosclerotic plaques in ApoE−/− mice
Source: Sci Rep. 2018 May 14;8:7530. doi: 10.1038/s41598-018-25942-3 (PMC5951920; doi:10.1038/s41598-018-25942-3)
Supplement: Supplementary file 1 — Supplementary information [file 41598_2018_25942_MOESM1_ESM.docx]

**SUPPLEMENTARY INFORMATION**

**Hyperglycemia does not affect tissue repair responses in shear stress-induced atherosclerotic plaques in ApoE-/- mice**

Sabrina Hsiung*^1^, Anki Knutsson^1^, Jenifer Vallejo^2^, Pontus Dunér^2^, Suvi E. Heinonen^3^, Ann-Cathrine Jönsson-Rylander^3^, Eva Bengtsson^2^, Jan Nilsson^2^ and Anna Hultgårdh-Nilsson^1^.

^1^Department of Experimental Medical Science, Lund University, Lund, Sweden

^2^Department of Clinical Sciences, Lund University, Malmoe, Sweden

^3^Heart Failure Bioscience, Cardiovascular and Metabolic Diseases, IMED Biotech Unit, AstraZeneca, Gothenburg, Sweden

*Corresponding author: Sabrina Hsiung
Department of Experimental Medical Science, BMC C12
SE-221 84 Lund, Sweden
Tel: + 46 46 2228576
Email: [Sabrina.Hsiung@med.lu.se](mailto:Sabrina.Hsiung@med.lu.se)


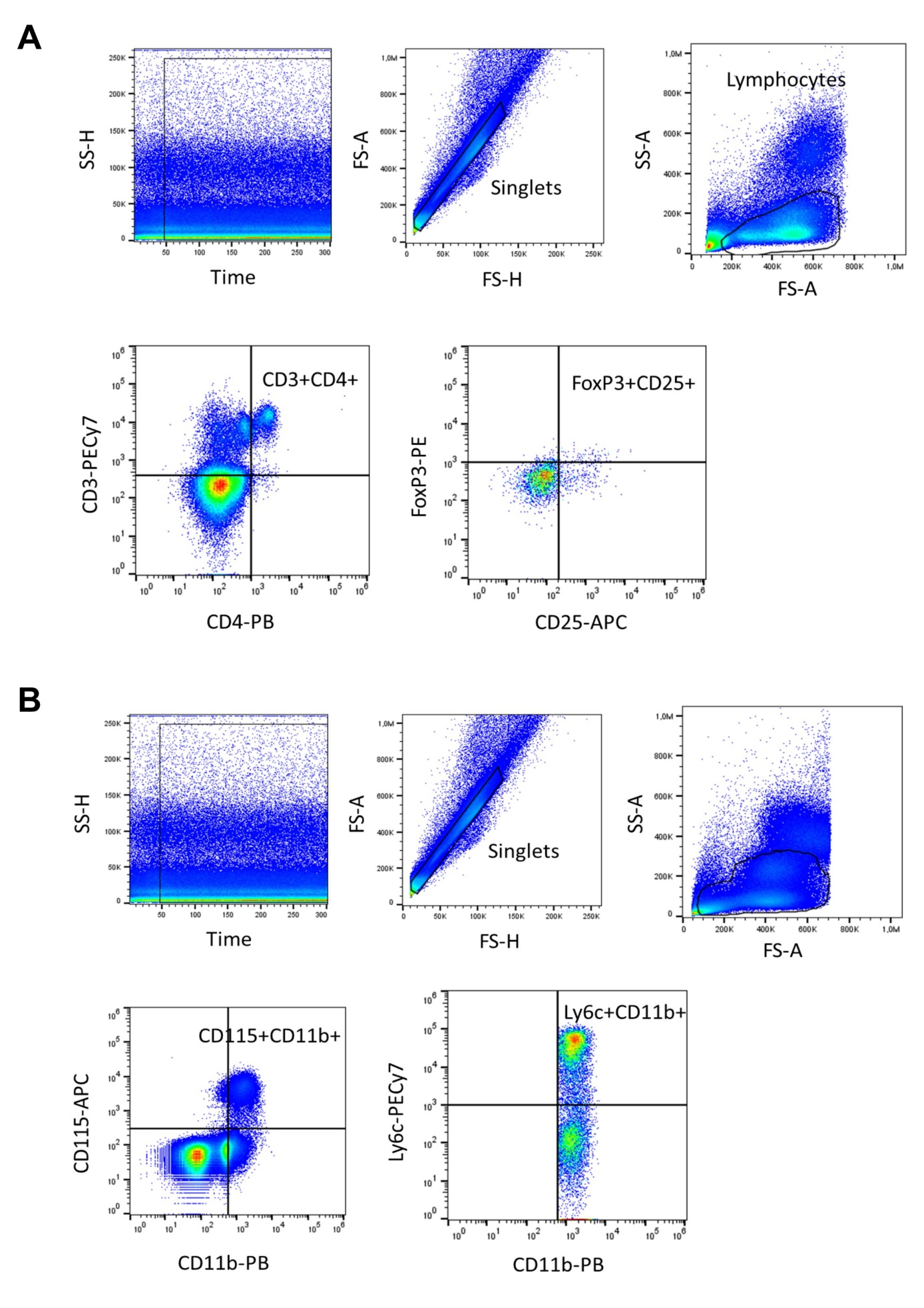


**Supplementary Figure S1. Flow cytometry analysis.** The gating strategy for flow cytometry analysis of (**A**) regulatory T-cells and (**B**) monocytes in ApoE-/-GK+/- and ApoE-/- mice.


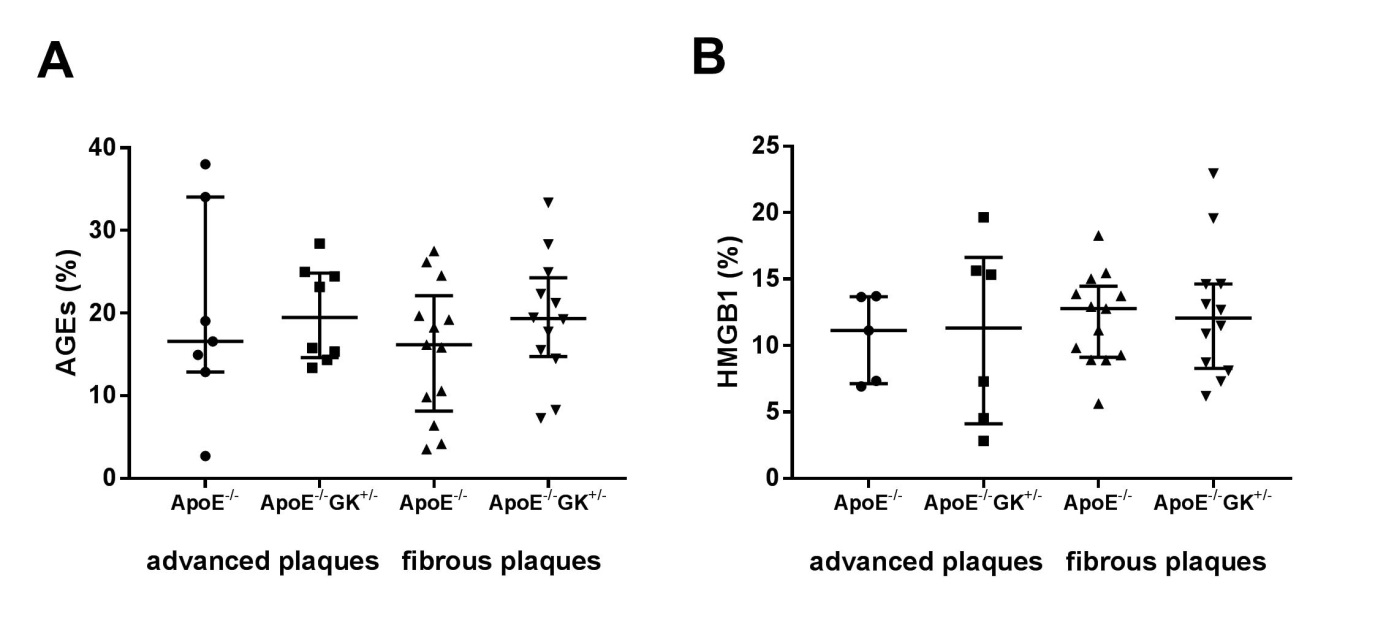


**Supplementary Figure S2. Advanced glycation end products and high mobility group box 1.** Elevated glucose levels did not affect the levels of AGEs (**A**) or HMGB1 (**B**) in the induced carotid lesions in either the ApoE-/-GK+/- mice or ApoE-/- mice.
